# Lightweight Detection and Adaptive Path Planning for Selective Hotan Rose Harvesting

**DOI:** 10.3390/s26092848

**Published:** 2026-05-02

**Authors:** Jijing Lin, Yuhang Yang, Baojian Ma, Zhenghao Wu, Bangbang Chen

**Affiliations:** 1 Department of Mechanical and Electrical Engineering, Xinjiang Institute of Technology, Aksu 843100, China; 2023274@xjit.edu.cn (J.L.); chenbangbang@st.xatu.edu.cn (B.C.); 2 College of Mechanical and Electrical Engineering, Shihezi University, Shihezi 832003, China; wuzhenghao@stu.shzu.edu.cn

**Keywords:** rose, lightweight recognition, path planning, selective harvesting

## Abstract

Selective harvesting of Hotan roses requires distinguishing between buds and blooms for different industrial uses. However, balancing detection accuracy and computational efficiency for edge deployment remains a challenge. This study proposes an integrated framework combining a lightweight detection model, Rose_YOLO, with an adaptive path-planning algorithm, the ROSE algorithm, to address these issues. The Rose_YOLO model optimizes the YOLOv8n architecture by incorporating the C2f-Faster-CGLU module and a Rose_Head detection head to enhance feature extraction while reducing redundancy. The ROSE algorithm integrates an improved genetic algorithm (GA) with a reciprocating search mechanism to dynamically optimize picking sequences based on scene complexity. Experimental results demonstrate that Rose_YOLO achieves a precision of 90.4% and a mAP@0.5 of 96.6% for blooms and a precision of 88.4% with a mAP@0.5 of 91.7% for buds. Compared to the baseline YOLOv8n, the model reduces parameters by 47.46% to 1.579 million, compresses the size to 3.19 MB, and lowers computational complexity to 4.6 GFLOPs. For path planning, the ROSE algorithm generates optimal paths with an average length of 2796.94 pixels, which is 73.1% shorter than the reciprocating algorithm and 51.6% shorter than the standard GA. Furthermore, it achieves an average runtime of only 7.33 ms, significantly outperforming traditional methods with respect to computational speed. In conclusion, the proposed framework achieves a superior balance between lightweight design and detection performance. The successful deployment on edge devices validates its effectiveness in providing real-time visual guidance and efficient path planning, offering a robust technical solution for the automated selective harvesting of roses in complex field environments.

## 1. Introduction

As a characteristic economic flower in Xinjiang, Hotan roses (commonly known as the “desert rose”) have important application value in food, cosmetics, and other industries. Hotan roses have two main growth states: flower and flower bud. Flowers are mainly used for processing essential oil, rose jam, and other products, while flower buds are commonly used for making scented tea. Both possess high economic value. However, the current picking of the Hotan rose still completely relies on manual labor, which severely restricts the large-scale cultivation of this crop. Due to the inconsistent flowering cycle, mechanized blind harvesting is infeasible, and selective harvesting is therefore required. Consequently, robotic selective harvesting technology is expected to bring a breakthrough for this industry. Among the key technologies, the recognition of Hotan roses and the planning of continuous picking represent critical technical challenges for realizing robotic harvesting.

As a prerequisite for robotic harvesting, precise recognition of Hotan roses’ flowers and buds is essential. While extensive research has focused on flower recognition, conventional image processing algorithms suffer from poor robustness in complex scenarios, failing to meet practical operational requirements [1,2]. To surmount the limitations of traditional visual recognition approaches, recent studies have integrated attention mechanisms and multi-scale feature fusion into deep learning architectures to boost model adaptability. For example, Zhang et al. [3,4] proposed several models based on Faster R-CNN and YOLO architectures to improve the recognition of safflower filaments. Additionally, Zhang’s team [5] devised a safflower filament neck localization framework combining improved particle swarm optimization (PSO) and a rotated bounding box algorithm, achieving 89.75% average picking-point localization accuracy in complex scenes. In parallel, Ma et al. [6] presented PointSafNet, an improved PointNet++ network for safflower picking-point recognition via point cloud data, which integrated spatial vector analysis and an oriented bounding box (OBB) algorithm to realize 90% localization accuracy. Nevertheless, these methods are effective only for single-stage recognition, whereas the dynamic morphological and color variations between rose flowers and buds during growth render single-stage visual detection inadequate for practical harvesting applications.

Recent years have witnessed increasing efforts to address the limitations of single-stage recognition by applying deep learning methods to the analysis of multiple floral growth stages. For instance, Shinoda et al. [7] developed the RoseTracker system, which integrates object detection and tracking techniques to achieve highly accurate counting of rose flowers and buds in a greenhouse. Zhao et al. [8] proposed an improved CR-YOLOv5s algorithm for detecting chrysanthemum buds and blooming flowers, achieving a mean average precision of 93.9%, which was 4.5% higher than that of the original YOLOv5s under complex backgrounds. Bai et al. [9] introduced an improved YOLO-based model that significantly improved the detection accuracy of flowers and fruits in strawberry seedlings by incorporating a Swin Transformer-based high-resolution prediction head and a GS-ELAN module, reaching an mAP of 92.1%. In addition, Ge et al. [10] proposed a picking-point localization method for safflower throughout the full harvesting period based on the SBP-YOLOv8s-seg network; through network structure optimization and a stage-wise localization strategy, their method significantly improved the recognition accuracy of safflower flowers and bolls in complex field environments. Furthermore, Zhang et al. [11] proposed a UAV-based method to classify chrysanthemum flowering stages (early, full, and late) by extracting canopy color features via Mask R-CNN and applying a color-ratio decision tree with a random forest model. Although these methods have made notable progress in multi-stage flower recognition, they still suffer from high model complexity and computational cost, which limits their deployment on mobile robotic platforms with constrained computing resources.

To address this challenge, recent studies have focused on improving network architectures to better balance the accuracy and efficiency of flower detection models. For example, Park et al. [12] proposed a lightweight YOLOv4-Tiny object detection system using circular bounding boxes to accurately classify the flowering stages of chrysanthemums, including full bloom, early bloom, and bud stage. Qi et al. [13] developed a lightweight convolutional neural network model, which enabled high-precision real-time detection of medicinal chrysanthemum buds in complex unstructured environments, such as varying illumination, occlusion, and overlap. In another study, Qi et al. [14] proposed TC-YOLO, a lightweight model based on feature fusion, which achieved an average precision (AP) of 92.49% and an inference speed of 47.23 FPS for detecting tea chrysanthemums during the flowering stage. In addition, Chen et al. [15] introduced a lightweight YOLO-SaFi safflower recognition model, which significantly reduced the number of parameters, computational cost, and model size of YOLOv8n by incorporating the StarNet backbone, designing the ELC convolution module, and adopting a lightweight detection head (Detect_EL). Furthermore, Yi et al. [16] proposed the lightweight flower detection model Light-FC-YOLO, which outperformed YOLOv8s in detection accuracy, with a 0.8% increase in mAP, while reducing the number of parameters by 39.0% and the model size by 27.2%.

In addition to network architecture optimization, model lightweighting can also be achieved through pruning and knowledge distillation. For instance, Fan et al. [17] enhanced YOLOv7 for marigold corolla detection by removing redundant layers, adopting DSConv, and applying pruning, thereby achieving a lightweight model. Similarly, Lyu et al. [18] proposed a litchi flower sex detection method based on YOLO-HPFD with multi-teacher feature distillation, which was successfully deployed on a low-power platform. In addition, Fatehi et al. [19] improved the performance of YOLOv9t for real-time detection of open Damask rose flowers via knowledge distillation while improving detection speed by 5.1 FPS and 1.8 FPS. These findings demonstrate that such strategies can substantially enhance the efficiency and accuracy of lightweight models in complex agricultural environments. Meanwhile, flower recognition technologies have been extended to other application scenarios, including flower counting and density estimation [20,21], as well as pollination and blossom thinning [22,23,24]. Although these studies provide valuable insights for lightweight flower recognition on mobile platforms, their applications have mainly focused on crops such as safflower, chrysanthemum, and marigold, with limited attention paid to the simultaneous recognition and analysis of both rose flowers and rose buds. Moreover, few studies have gone beyond recognition itself to investigate the planning problem of continuous flower harvesting.

Beyond flower recognition, the coordinated optimization of continuous picking path planning represents another major challenge. It should be emphasized that accurate recognition constitutes merely the initial step toward the practical deployment of harvesting robots; the manner in which continuous picking paths are planned based on recognition results—while aiming to avoid redundant operations—directly determines harvesting efficiency [25,26]; however, studies on continuous picking sequence planning after the recognition of terminal flower targets are still in their infancy. Most existing studies treat target recognition and path planning as two independent functional modules, and the coordination mechanism between them remains insufficiently explored. For example, Ge’s team [27] proposed an improved ant colony optimization method for the picking path planning of terminal safflower flowers. However, safflower recognition was not addressed in that study. Similarly, Zhang et al. [28] proposed a dual-arm safflower picking path-planning method using an improved ant colony algorithm with dynamic-weight heuristic, K-means partitioning, and 2-OPT local search, enhancing efficiency and stability, but still did not perform path planning based on flower recognition results. On the other hand, Wang et al. [29] formulated the task as a three-dimensional traveling salesman problem and combined a Pointer Network with the Actor–Critic (AC) algorithm in deep reinforcement learning for dynamically planning the picking sequence of safflowers with different quantities and spatial distributions. In addition, Ma et al. [30] proposed a lightweight marigold corolla recognition model; when combined with an improved ant colony algorithm, the path-planning time was reduced to 2.2 s. Although considerable progress has been made in terminal flower recognition and path planning, existing studies have mainly focused on crops such as chrysanthemum and safflower, making it difficult to adapt their methods to the more complex harvesting requirements of roses, which must be picked according to different developmental states. In particular, rose plants exhibit dense growth patterns, flowers that are often adhesive or overlapping, and small, highly clustered buds—characteristics that hinder the direct application of existing path-planning methods for picking. Therefore, a coupled framework that integrates lightweight recognition with continuous path planning is urgently required to simultaneously ensure high recognition accuracy and planning efficiency, offering a new technical pathway toward automated rose harvesting.

To this end, this study proposes a coupled framework integrating a lightweight recognition model for rose flowers and flower buds with a continuous path-planning method:

(1) In this work, a lightweight deep learning network is proposed for efficient recognition, achieving high detection accuracy while substantially reducing model complexity and computational cost. This makes the approach particularly suitable for deployment on embedded platforms.
(2) Based on lightweight recognition of rose flowers and flower buds, this study proposes an adaptive continuous picking path-planning algorithm. By integrating an improved genetic algorithm with a reciprocating search mechanism, an efficient global path-planning strategy is developed to generate an optimal picking path that achieves a favorable trade-off between operational efficiency and path continuity.

## 2. Materials and Methods

### 2.1. Image Acquisition and Data Processing

**Figure 1:**
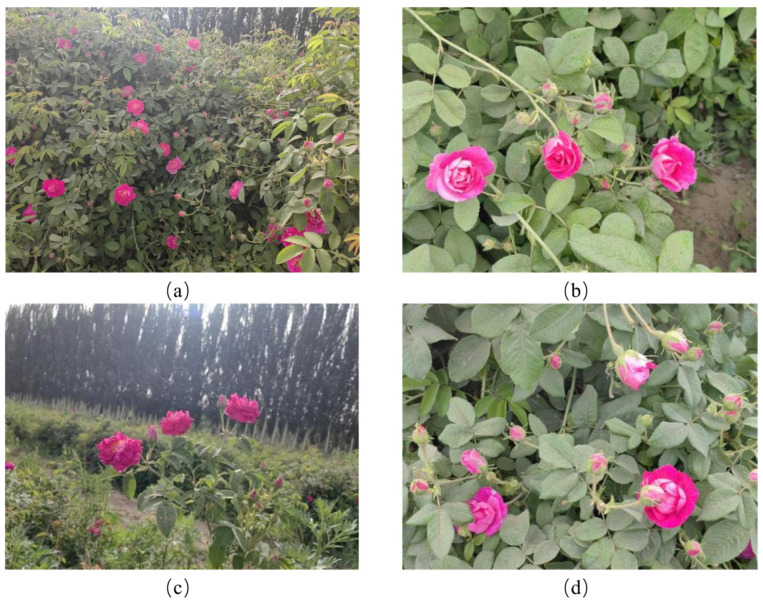
Diverse contexts: (**a**) rose data acquisition; (**b**) front lighting; (**c**) backlighting scenario; (**d**) occlusion.

In this study, roses cultivated in the Hotan region of Xinjiang, China, were used as the research object. A high-quality rose object detection dataset was constructed based on 2897 high-resolution images captured by a Huawei P20 mobile device (Huawei Technologies Co., Ltd., Shenzhen, China), with a resolution of 4160 × 3120 pixels (Figure 1). The data collection covered the flower bud stage and full blooming stage of rose growth, during which the number of buds under natural growth conditions is typically greater than that of blooming flowers. Various complex scenarios were also captured, including severe leaf occlusion, dense target overlap, and drastic illumination changes.

To improve annotation efficiency while ensuring annotation quality, a model-assisted semi-automatic annotation strategy was proposed and implemented. Specifically, 200 images were randomly sampled from the raw dataset and manually annotated with high precision to form an initial training set, accompanied by corresponding XML-formatted annotation files; subsequently, YOLOv8n was trained iteratively on this subset to obtain the optimal weight file (best.pt). To enhance cross-platform compatibility and inference efficiency, the model weights were converted into ONNX format (best.onnx) and integrated into the X-AnyLabeling annotation tool, enabling automated detection and preliminary annotation for the remaining images. Following automated annotation, the results were rigorously reviewed by three domain experts in a cross-validation manner, with particular attention paid to correcting missed detections, false positives, and bounding box localization errors to ensure annotation accuracy and consistency. The final dataset comprises 2897 finely annotated images, which were randomly partitioned into training, validation, and test sets in an 8:1:1 ratio for subsequent model training and performance evaluation. To prevent data leakage and avoid over-optimistic evaluation, dataset partitioning was performed at the batch level rather than at the level of individual images. Specifically, images acquired from the same plant or captured within short temporal intervals were treated as a single unit during the splitting process, thereby ensuring that highly similar samples did not appear concurrently in both the training and test sets.

### 2.2. Overview of the Method

**Figure 2:**
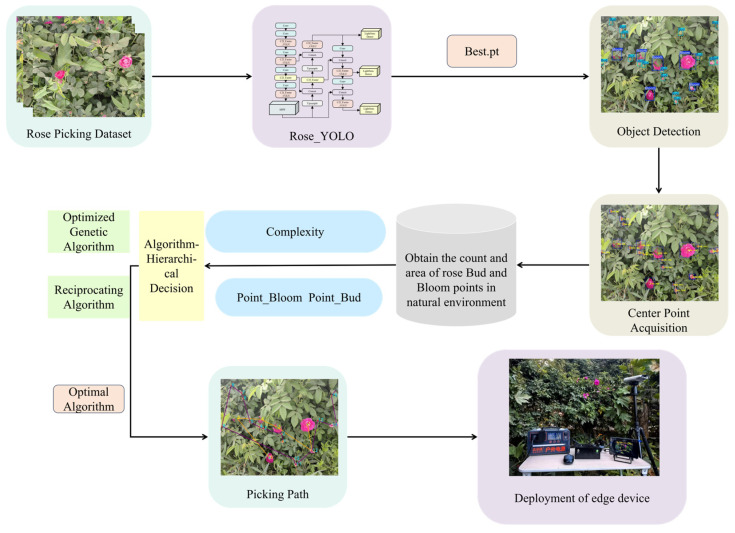
Path planning for rose blooms and buds based on lightweight models.

This study proposes a path-planning method tailored for automated rose harvesting in the Hotan region of Xinjiang, with the overall workflow illustrated in Figure 2. The method integrates an improved object detection model and an adaptive path optimization algorithm to establish an end-to-end processing pipeline from raw image input to optimal picking path output. Specifically, using raw rose images as input, the proposed Rose_YOLO model—built upon a modified YOLOv8n architecture—first processes the input data. The integration of the C2f-Faster-CGLU (Convolutional Gated Linear Unit) module and the Rose_Head structure enables the model to retain its lightweight characteristics while achieving substantial improvements in both detection accuracy and robustness when identifying rose flowers and flower buds. The model outputs object detection results with class labels and bounding boxes, enabling accurate recognition of roses at different growth stages. On this basis, the system further extracts the geometric center points of each detected target, marked as Point_Bloom and Point_Bud, respectively, which serve as candidate waypoints for subsequent path planning. Based on the obtained set of center points, a hierarchical decision-making strategy is adopted for path optimization, and the ROSE (Rose Optimization Search and Evaluation) path-planning algorithm is developed by combining the genetic algorithm and the reciprocating search mechanism: the genetic algorithm is responsible for the global search of the optimal path sequence, while the reciprocating search optimizes the movement efficiency in the local area. The synergy of the two strategies achieves the unification of path length minimization and operational continuity.

### 2.3. Selection and Optimization of the Baseline Model

In the field of single-stage object detection, the YOLO series has become one of the most representative and widely adopted frameworks owing to its superior inference efficiency and real-time detection capability. Among these models, YOLOv8n achieves a favorable balance among model complexity, computational cost, and detection accuracy, making it particularly suitable for deployment scenarios with limited resources and stringent real-time requirements. The architecture of YOLOv8n mainly consists of four functional components: input processing, backbone, neck, and detection head. Specifically, the input module enhances the generalization ability of the model through diverse data augmentation strategies; the backbone adopts an efficient feature extraction mechanism that preserves rich deep semantic information while substantially reducing computational burden; the neck aggregates multi-scale feature maps through a hierarchical feature fusion structure, thereby improving the model’s perception of objects with scale variation; and the detection head performs accurate classification and localization while maintaining high inference speed, thus achieving a reasonable trade-off between detection accuracy and efficiency. The YOLOv8 family provides several model variants, including *n*, *s*, *m*, *l*, and *x*, among which YOLOv8n has the lowest parameter count and computational overhead, demonstrating a clear speed advantage in constrained environments such as edge computing platforms. Considering that the rose detection task addressed in this study imposes explicit requirements on lightweight deployment and real-time performance, YOLOv8n was selected as the baseline detection model.

#### 2.3.1. Rose_YOLO Model

**Figure 3:**
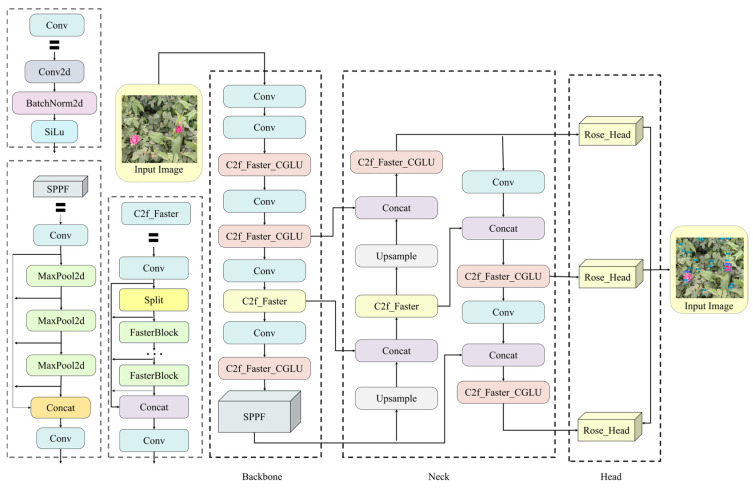
The framework of Rose_YOLO model.

In this study, a lightweight detection model, termed Rose_YOLO, is proposed for the detection of Hotan rose flowers and buds in natural scenes. Built upon the YOLOv8 baseline architecture, the proposed model significantly improves computational efficiency while reducing memory consumption and model size, without compromising detection accuracy, thereby making it more suitable for deployment on edge computing devices. To address the limitations of the original YOLOv8 in recognizing small-scale rose targets under complex backgrounds, as well as the redundancy in feature representation, this study systematically optimizes and redesigns three key components of the network, namely the backbone, feature fusion module, and detection head. The overall architecture of the proposed network is illustrated in Figure 3.

First, with respect to the backbone design, this study retains the original hierarchical feature extraction architecture of YOLOv8 while focusing on the reconstruction and optimization of its key feature extraction units. Specifically, a lightweight C2f_Faster_CGLU module with a gating mechanism is introduced to replace the original C2f module and is deployed at three critical stages, namely layers 2, 4, and 8. This module adopts a Convolutional Gated Linear Unit (CGLU) structure, which achieves feature decoupling along the channel dimension, thereby effectively reducing computational complexity while enhancing the nonlinear representation and cross-stage feature modeling capability of the network. Meanwhile, the essential downsampling convolution layers (layers 0, 1, 3, 5, and 7) and the Spatial Pyramid Pooling-Fast (SPPF) module are preserved to ensure efficient capture of multi-scale contextual information. Through this design, the backbone network is effectively made lightweight while maintaining its fundamental feature representation capacity.

Second, in the design of the feature-fusion neck, this study preserves the classical bidirectional fusion mechanism of YOLOv8, which combines bottom-up and top-down pathways to enable cross-scale feature interaction through upsampling, downsampling, and feature concatenation operations. On this basis, a hierarchically optimized feature enhancement strategy is proposed, in which the original C2f structures are replaced by the improved C2f_Faster and C2f_Faster_CGLU modules. Specifically, the C2f_Faster module is deployed at layer 12 to improve forward inference efficiency, whereas the C2f_Faster_CGLU modules are introduced at layers 15, 18, and 21 to strengthen the selective representation of high-level semantic features through a gated linear unit mechanism. This hierarchical design not only improves the fusion efficiency of multi-scale features but also suppresses the propagation of unnecessary noise through gated feature selection, thereby substantially enhancing the extraction of discriminative features for small bud targets in complex images.

Finally, to address the issues of excessive parameter count and high computational complexity in the YOLOv8 detection head, this study proposes a novel Rose_Head based on a lightweight shared-convolution architecture. Departing from the conventional paradigm that employs independent convolutional operations across different scales, the proposed module reconstructs the feature extraction process by introducing a unified weight-sharing strategy for multi-scale feature maps (P3, P4, and P5). Through channel unification and shared kernel design, this approach achieves efficient parameter reduction, thereby enhancing detection accuracy while substantially eliminating redundant computational overhead. In summary, by jointly optimizing the three core components of the baseline YOLOv8 framework—namely the backbone, neck, and detection head—the proposed Rose_YOLO model attains an effective balance among detection accuracy, model size, and memory consumption, demonstrating strong potential for real-time rose recognition in resource-constrained scenarios.

#### 2.3.2. C2f-Faster-CGLU Module

To address the feature extraction redundancy and low computational efficiency of the Bottleneck structure in the conventional C2f module under complex scenarios, this study proposes an improved scheme based on the collaborative architecture of FasterBlock and CGLU to replace the original serial structure. This scheme systematically reconstructs the feature processing pipeline from the perspectives of channel utilization efficiency and computational logic. Specifically, FasterBlock serves as the core fundamental component and, while preserving the necessary floating-point operations to maintain sufficient feature extraction depth, innovatively introduces a partial convolution (PConv) mechanism. More specifically, this mechanism extracts critical features from only a subset of the channels in the input feature map. Under the premise of keeping the total number of channels unchanged, we set the separation ratio $r=c_{p}/c=1/4$, where $c_{p}$ denotes the number of channels participating in local computation. This strategic reduction means the computational burden of PConv is merely 1/16 of that of a standard convolution. The theoretical speed-up ratio R=16.

**Figure 4:**
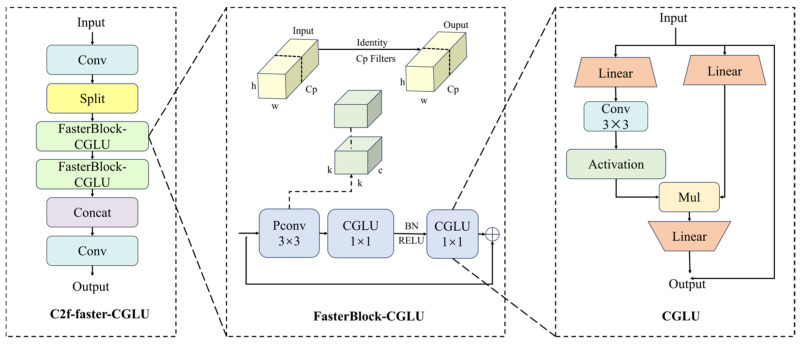
Structure of the C2f-Faster-CGLU module.

This design not only accommodates the feature representation requirements of complex scenes but also effectively suppresses redundant feature interactions, thereby improving overall computational efficiency. A schematic illustration of the underlying principle is shown in Figure 4. To further enhance feature representation capability, this study deeply integrates the CGLU module with FasterBlock to construct a FasterBlock-CGLU module. In the optimization of the CGLU module, particular emphasis is placed on improving the gating mechanism by introducing a 3×3 depthwise deformable convolution before the activation function in the GLU gating branch. The gating function is defined in Equation (1):

(1) $$\mathrm{GLU}(x)=(Wx_{1})⊙\sigma (Wx_{2})$$

In this equation, W denotes the learnable weight matrix (specifically the depthwise deformable convolution kernel in this context); $x_{1}$ and $x_{2}$ represent the input feature partitions for content and gating control, respectively; σ signifies the Sigmoid activation function; and ⊙ indicates element-wise multiplication.

This mechanism aligns with the design principle of gated channel attention and further evolves it into a gated channel attention mechanism based on nearest-neighbor feature correlation. Through dynamically generated gating signals, this mechanism enables effective feature selection and substantially improves the specificity and discriminative power of feature representation. By directly replacing the original Bottleneck structure in C2f with this module, the C2f-Faster-CGLU module is obtained. Within this architecture, the parameter reduction and computational acceleration brought by PConv work synergistically with the structural compatibility between the batch normalization layers and adjacent convolutional layers in FasterBlock, allowing the model to achieve an effective balance between lightweight design and detection performance while maintaining controllable accuracy degradation, thereby fundamentally overcoming the inherent efficiency bottleneck of the conventional C2f module.

#### 2.3.3. Rose_Head Detection Head

**Figure 5:**
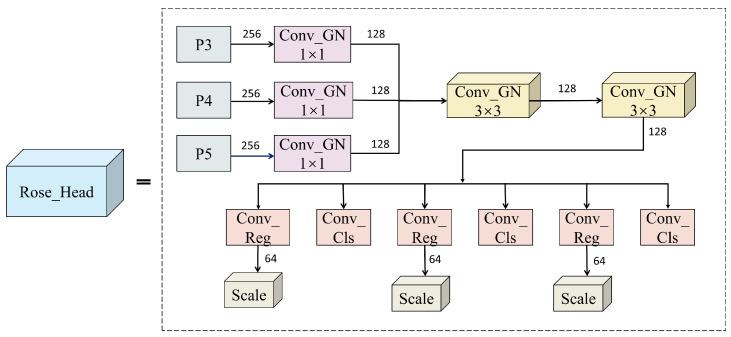
Lightweight Rose_Head detection head.

This study proposes a lightweight detection head termed Rose_Head (Figure 5), with its core innovation lying in the introduction of a shared convolution mechanism to reformulate the feature extraction process. Unlike conventional detection heads that perform independent convolution operations on multi-scale feature maps, the proposed architecture unifies feature extraction across the P3, P4, and P5 layers via a weight-sharing strategy. The shared feature extraction process is formulated in Equations (2) and (3):

(2) $$Y_{i}^{1}=\mathrm{SiLU}(\mathrm{GN}({\mathrm{Conv}}_{3\times 3}(X_{i};W^{1},b^{1})))$$

(3) $$Z_{i}=\mathrm{SiLU}(\mathrm{GN}({\mathrm{Conv}}_{3\times 3}(Y_{i}^{1};W^{2},b^{2})))$$

Here, let $X_{i}$ denote the input feature map at level i∈{3,4,5}, while $Y_{i}^{1}$ and $Z_{i}$ represent the outputs of the first and second convolutional blocks, respectively. The parameters $W^{1},b^{1}$ and $W^{2},b^{2}$ correspond to the learnable weights and biases of the shared 3 × 3 kernels. Additionally, ${\mathrm{Conv}}_{3\times 3}$, GN, and SiLU denote the convolution operation, Group Normalization, and the Sigmoid Linear Unit activation function, respectively.

This design effectively reduces the number of model parameters and mitigates computational redundancy. Furthermore, it enhances the semantic consistency of convolutional kernels across different scales, thereby yielding more robust feature representations. To accommodate the substantial morphological variations of rose plants during the transition from the bud stage to full bloom, a streamlined decoupled structure is adopted, where the regression and classification outputs are derived separately. In this architecture, the P3 layer preserves high-resolution, fine-grained texture information; the P4 layer captures mid-scale morphological contour features; and the P5 layer retains low-resolution, global semantic context. Such a hierarchical design enables the model to efficiently extract features from roses at different flowering stages. Notably, the high-resolution P3 features are effectively leveraged to enhance the perception of small-scale targets (e.g., early-stage buds), thereby alleviating missed detections caused by scale variation. Additionally, for the regression task, continuous distance values are obtained by calculating the expectation of the distribution. Owing to the shared-kernel design, the proposed Rose_Head avoids redundant convolution operations, substantially reducing the computational burden and memory overhead while maintaining high detection accuracy. As a result, the proposed method achieves an optimal balance between model lightness and detection performance.

### 2.4. Overall Framework for Harvesting Path Planning

The spatial heterogeneity of rose morphology and flower distribution poses substantial challenges to efficient harvesting path planning. Traditional algorithms operating in such unstructured environments are prone to falling into local optima while also suffering from high computational cost and limited generalization capability. To address these issues, this study aims to achieve a mechanism synergy between search performance and computational overhead. On the premise of ensuring the geometric feasibility of the path, the system employs a hierarchical decision-making mechanism to dynamically balance the energy consumption metrics of the path with the real-time response capability of the algorithm for different task scenarios.

**Figure 6:**
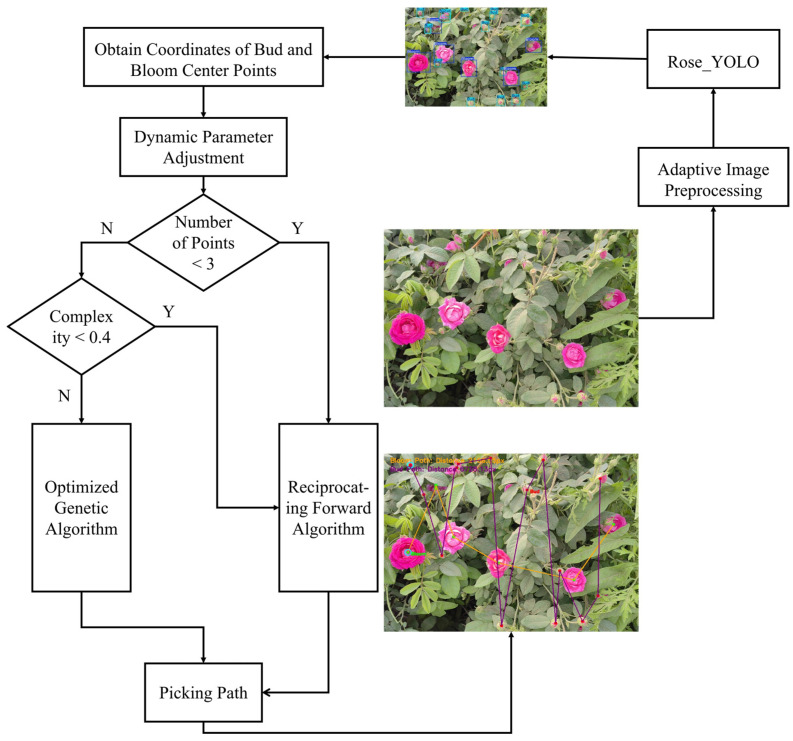
Framework of adaptive harvesting path planning.

The overall workflow begins with image-adaptive preprocessing to enhance and normalize the input images, thereby improving the robustness of subsequent target detection (Figure 6). The preprocessed images are then fed into the Rose_YOLO model for high-precision detection of buds and flowers, through which the spatial center coordinates of the targets are obtained. Upon acquiring target location information, the system formulates the path-planning task as a minimization problem of total travel distance (energy consumption). The mathematical expression for this objective is presented in Equation (4):

(4) $$minF_{2}(p)=\sum_{i=1}^{n-1}d(p_{i},p_{i+1})+d(p_{n},p_{1})$$

where $p_{i}$ denotes the spatial coordinates of the i th detected flower or bud target, and n represents the total number of targets to be harvested. The term $d(p_{i},p_{i+1})$ signifies the Euclidean distance between two consecutive targets in the sequence, while $d(p_{n},p_{1})$ represents the return distance from the final target back to the starting point, thereby closing the harvesting loop.

To achieve this objective under real-time constraints, a hierarchical decision-making mechanism is introduced. Specifically, when the system identifies a scenario as simple, it directly applies a reciprocating forward traversal strategy. This strategy simplifies the path-planning problem into a sorting operation, achieving an optimal time complexity of O(nlogn) and ensuring millisecond-level response speeds. Conversely, for scenarios identified as complex, the system employs an improved genetic algorithm (GA) for global path optimization. By constraining the population size (N) and the number of generations (G) to small constants to fit within the real-time window, the GA searches for the optimal harvesting path with a controllable complexity of $O(n^{2})$, minimizing travel distance and time to ultimately generate the optimal path. The pseudocode and implementation details are presented in Algorithm 1.

**Figure 7:**
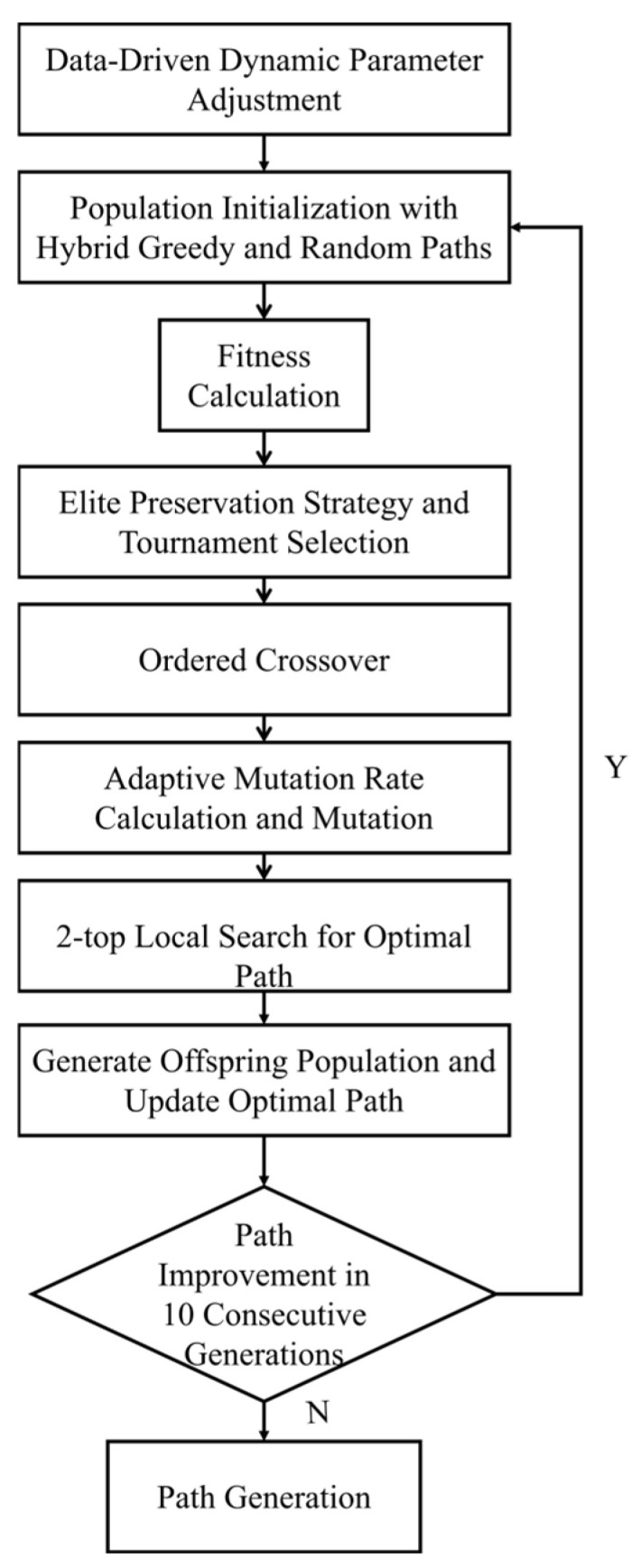
Optimization process of the genetic algorithm.

In the optimization process of the genetic algorithm, this study first introduces, at the image preprocessing stage, a pixel-count-based dynamic scaling mechanism and a secondary memory calibration strategy (Figure 7). When combined with the Lanczos interpolation algorithm, these mechanisms achieve an effective balance between detection accuracy and inference efficiency. Furthermore, a hybrid optimized genetic algorithm is designed, in which the population is initialized through a combination of greedy initialization and random initialization to improve both the quality and diversity of the initial solutions. To ensure the stable inheritance of high-quality genes, a collaborative mechanism integrating elite retention and tournament selection is adopted; meanwhile, an adaptive mutation-rate adjustment strategy is introduced, whereby the mutation probability decays linearly with the number of evolutionary generations, thus balancing global exploration capability and local convergence performance. On this basis, the algorithm incorporates a 2-opt local search strategy, which effectively removes path crossings and accelerates convergence, thereby significantly shortening the iterative cycle of conventional genetic algorithms. To reduce unnecessary computational resource consumption, an intelligent termination mechanism is further developed, allowing the iteration process to stop early when no path improvement is observed over ten consecutive generations. In addition, a dynamic parameter adjustment strategy is proposed to adaptively configure the population size and the number of iterations according to the number of detected target points, and a scene complexity evaluation model based on flower density and average distance is constructed. Through a decision-tree-based switching mechanism between the reciprocating traversal algorithm and the genetic optimization algorithm, the proposed method enables rapid response in low-complexity scenarios and high-quality path planning in high-complexity scenarios, thereby comprehensively enhancing the robustness and real-time performance of the algorithm in open-field environments.
**Algorithm 1.** The pseudocode for the genetic algorithm’s computational procedure.Input: Rose Detection Results (Flower Category; set of central coordinates of flowers); scene areaOutput: Optimal path-planning algorithm; Planned path*n* = len(Points)   # Count the number of flowers*ρ* = *n*/Area       # Calculate flower density (number of flowers per unit area)*d* = Average Euclidean Distance*c* = *w*_time * (*ρ*/(1 + *ρ*)) + *w*_distance * (*d*/(1 + *d*))   # Scene Complexity; Weight*w*_time = 0.6, *w*_distance = 0.4if *n* < 3:                  selected_alg = Reciprocating_Algorithm      elif *n* > 20:                  selected_alg = Genetic_Optimized_Algorithmelse:                  initial_alg = Reciprocating_Algorithm if *c* <= 0.4 else Genetic_Optimized_Algorithm                        if Category == “Bud” and *ρ* > 0.5:                               selected_alg = Genetic_Optimized_Algorithm                        else:selected_alg = initial_alg

### 2.5. Experimental Environment and Parameter Settings

To ensure experimental reproducibility and computational efficiency, all experiments were conducted on a high-performance workstation equipped with an Intel Core i9 processor (Intel Corporation, Santa Clara, CA, USA) and a single GPU featuring 16 GB, operating under the Windows 10 system. The software environment was uniformly configured within a virtual environment established via Anaconda, utilizing Python 3.9 as the programming language and PyTorch 2.2.2 as the deep learning framework. To fully leverage the parallel computing capabilities of the GPU, CUDA 12.1 and cuDNN 11.3 were integrated into the software stack. Regarding the training strategy, all models were trained from scratch without employing ImageNet-pretrained weights, adhering to an end-to-end training paradigm throughout the experimental process. The stochastic gradient descent (SGD) algorithm was adopted as the optimizer, with a momentum of 0.9, a batch size of 32, and an initial learning rate of 0.01. Each model underwent training for 300 epochs to ensure adequate parameter convergence and stable performance.

### 2.6. Model Evaluation Indicators

In this study, model detection performance was systematically evaluated using three metrics: precision (P), recall (R), and mean average precision at an intersection over union threshold of 0.5 (mAP@0.5). To quantify model complexity and lightweight characteristics, model size, number of parameters, and floating-point operations (GFLOPs) were adopted as the primary indicators. For the performance evaluation of the path-planning algorithm, an assessment framework was established based on three key metrics: number of target points detected, average runtime, and average path length.

## 3. Results and Analysis

### 3.1. Ablation Experiment

To systematically evaluate the contribution of each key component in the proposed Rose_YOLO model to the overall performance, a series of ablation experiments was designed and conducted, and the results are presented in Table 1. Using YOLOv8n as the baseline model, the three proposed modules, namely C2f-faster, C2f-faster-CGLU, and Rose_Head, were introduced sequentially, and their effectiveness was analyzed from the two perspectives of model lightweighting and detection accuracy. First, compared with the baseline model, introducing the C2f-faster module alone reduced the model size from 5.94 MB to 4.62 MB, the number of parameters from 3.006 million to 2.301 million, and the GFLOPs from 8.1 to 6.3, achieving a significant compression of approximately 22%. Meanwhile, the mAP@0.5 for the Bloom category decreased by only 0.3 percentage points, and that for the Bud category decreased by 1.5 percentage points, indicating that this module substantially reduced model complexity while exerting only a minor impact on detection accuracy, thereby demonstrating excellent lightweight characteristics. Furthermore, after incorporating the CGLU module on the basis of C2f-faster, the model size was further reduced to 4.44 MB, the number of parameters decreased to 2.223 million, and the GFLOPs dropped to 6.2. Although the mAP@0.5 for the Bloom category slightly declined to 95.5% and the recall (R) for the Bud category exhibited slight fluctuations, the remaining key metrics showed an overall improving upward trend, among which the precision (P) and mAP@0.5 for the Bud category increased to 89.8% and 91.1%, respectively. These results indicate that the CGLU module effectively alleviates accuracy degradation by enhancing nonlinear interactions among feature channels while maintaining computational efficiency. Finally, after the full introduction of the Rose_Head module, the model achieved extreme lightweight performance, with the model size reduced to only 3.19 MB, the number of parameters compressed to 1.579 million, and the GFLOPs decreased to 4.6, corresponding to an overall compression rate of more than 46% relative to the baseline model. Notably, the mAP@0.5 values for the Bloom and Bud categories recovered to 96.6% and 91.7%, respectively, demonstrating improved detection accuracy under highly compressed model conditions. This result validates that the Rose_Head module effectively compensates for the performance loss caused by lightweight design by strengthening multi-scale feature fusion capability.

In summary, the C2f-faster module primarily contributes to the lightweight design of the backbone network; C2f-faster-CGLU further enhances feature representation capability while maintaining high computational efficiency; and Rose_Head, serving as a critical accuracy compensation mechanism, significantly improves detection accuracy under substantial compression conditions. Through the synergistic effect of these three components, the proposed Rose_YOLO model achieves a 46% compression rate while maintaining, or even surpassing, the baseline performance in detecting key categories. These results fully demonstrate that the proposed model attains an excellent balance between computational efficiency and detection performance.

### 3.2. Comparative Experiments Among Different Models

To evaluate the detection performance of the proposed Rose_YOLO model for different growth states of Hotan rose in natural environments, namely Bloom and Bud, this study systematically compared the performance of five models—YOLOv6n, YOLOv8n, YOLOv10n, YOLOv11n, and Rose_YOLO—under a unified experimental environment and dataset configuration. The results are presented in Table 2.

In terms of detection accuracy, Rose_YOLO achieved a precision of 90.4%, a recall of 91.6%, and an mAP@0.5 of 96.6% for the Bloom category, while for the Bud category, the corresponding values were 88.4%, 82.3%, and 91.7%, respectively. Although its performance on certain individual metrics was slightly lower than that of baseline models such as YOLOv6n and YOLOv11n, its overall detection accuracy remained highly competitive with current mainstream lightweight models, thereby confirming its effectiveness and robustness in target recognition tasks.

More importantly, Rose_YOLO exhibited significant advantages in model lightweighting. Compared with YOLOv6n, YOLOv8n, YOLOv10n, and YOLOv11n, the proposed model reduced storage size by 61.43%, 46.30%, 41.68%, and 39.24%; parameter count by 62.69%, 47.46%, 30.29%, and 38.84%; and computational complexity (GFLOPs) by 61.02%, 43.21%, 29.23%, and 26.98%, respectively. Specifically, Rose_YOLO requires only 3.19 MB of memory, contains approximately 1.579 million trainable parameters, and incurs only 4.6 GFLOPs during inference—all metrics substantially outperforming existing mainstream lightweight YOLO variants.

Although Rose_YOLO did not achieve the absolute best performance across all accuracy metrics, it achieved substantial reductions in model size, parameter count, and computational complexity while maintaining high detection performance, demonstrating excellent lightweight characteristics and strong potential for embedded deployment. These findings provide reliable technical support for the practical deployment of rose growth-state recognition models in natural environments.

**Figure 8:**
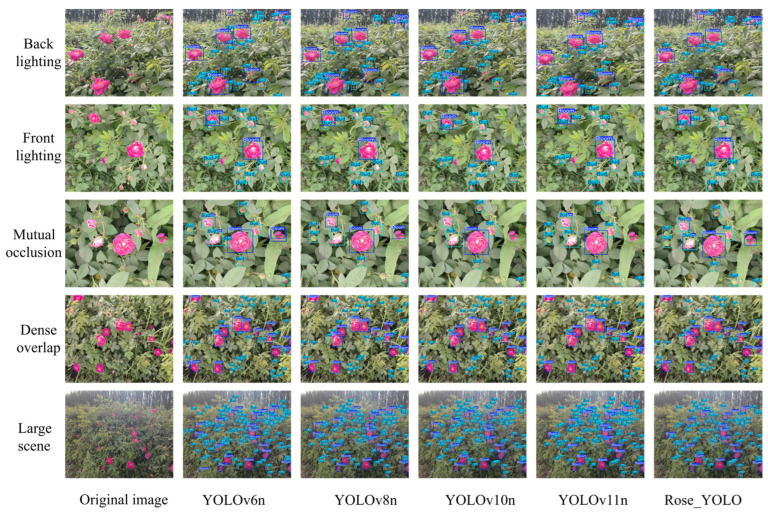
Detection results of different models.

In this study, comparative experiments were conducted on different versions of YOLO object detection models under multiple complex scenarios to systematically evaluate their practical performance. To comprehensively investigate model robustness and detection accuracy under challenging conditions such as illumination variation, object occlusion, and high-density distribution, a test set covering five representative and challenging scenarios was constructed, including backlighting, front lighting, mutual occlusion, dense overlap, and large-scale scenes, as illustrated in Figure 8.

Under backlighting conditions, experimental results demonstrate that Rose_YOLO achieves superior feature representation capabilities compared to other YOLO-based models, which are prone to missed and false detections—especially in low-contrast regions where accurate target recognition is challenging. This finding indicates that Rose_YOLO substantially enhances detection robustness under low-illumination conditions. Under front-lighting conditions, although all compared models achieve relatively stable target recognition, Rose_YOLO exhibits clear advantages in localization accuracy and redundant bounding box suppression. To address the issue of mutual occlusion among targets, Rose_YOLO incorporates a context-aware mechanism and attention enhancement modules, effectively improving the recognition of partially occluded objects and significantly reducing both false positive and false negative rates. In densely overlapping scenarios, the model mitigates target adhesion through an optimized multi-scale feature fusion architecture, yielding more precise detection performance. Furthermore, when processing large-scale scene images, Rose_YOLO maintains high detection efficiency while demonstrating superior global information modeling and long-distance target capture capabilities, with overall performance surpassing that of existing benchmark models.

**Figure 9:**
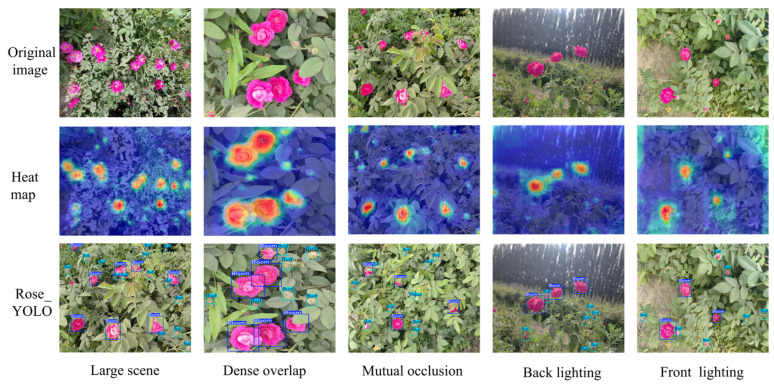
Detection heatmap of the Rose_YOLO model.

To systematically evaluate the object detection performance of Rose_YOLO in complex natural scenes, this study conducted a visual comparative analysis from three perspectives—original images, attention heatmaps, and detection results—under different representative environmental conditions, as shown in Figure 9. In large-scale scenes, Rose_YOLO was able to accurately identify discretely distributed rose targets, and the heatmaps revealed that the model concentrated primarily on the central regions of the flowers, demonstrating its strong global receptive field and spatial localization capability. In densely overlapping scenes, the model still achieved precise discrimination and accurate bounding-box localization even when flowers were closely adjacent, highlighting its excellent local discrimination and target separation ability. Under mutual occlusion conditions, although some flowers were partially obscured by leaves to varying degrees, the model was still able to accurately infer the locations of the occluded targets by leveraging contextual semantic information, indicating strong structural robustness. In backlit environments, where intense background illumination significantly reduced the contrast of foreground targets, Rose_YOLO achieved stable detection under low-contrast conditions through an enhanced response mechanism for features in weakly illuminated regions. Under front-lighting conditions, where illumination was uniform and target features were clear, the model not only achieved high-precision bounding-box localization but also effectively suppressed false detections. Taken together, these results demonstrate that Rose_YOLO exhibits outstanding object detection performance across various representative natural scenes, particularly showing remarkable robustness and adaptability in challenging tasks involving occlusion, illumination variation, and high-density target distribution.

### 3.3. Results of Different Algorithmic Strategies

To validate the effectiveness of the ROSE algorithm in path-planning tasks, this study conducted a systematic comparative experiment on a validation set consisting of 288 rose images collected in natural environments. Under unified experimental conditions, the performance of the proposed ROSE algorithm was compared with three representative path-planning methods, namely the reciprocating algorithm, ant colony algorithm, and genetic algorithm, and the results are presented in Table 3. The experimental results show that although the reciprocating algorithm incurred the lowest computational cost, with a processing time of only 0.000205 s per run, it produced a path length as high as 10,411.54 pixels, indicating a clear deficiency in path optimization capability. The genetic algorithm and ant colony algorithm achieved path lengths of 5778.46 pixels and 3827.24 pixels, respectively; however, their computation times reached 0.219869 s and 1.980572 s, respectively, making them less suitable for applications with stringent real-time requirements. In contrast, the proposed ROSE algorithm achieved the best balance between average runtime and average path length, with a computation time of only 0.00733 s and a path length as low as 2796.94 pixels. Compared with the reciprocating algorithm, ROSE reduced the path length by 73.1%; compared with the ant colony algorithm, its computation time was only 0.37% of that required by the latter. Moreover, relative to the genetic algorithm, ROSE shortened the path length by 51.6% while reducing computation time to only 3.33% of that of the genetic algorithm, fully demonstrating its superior optimization efficiency. Overall, the ROSE algorithm effectively reconciles the trade-off between computational efficiency and path quality, exhibiting outstanding comprehensive performance and making it particularly suitable for path-planning tasks that demand both high real-time performance and high-quality solutions.

### 3.4. Deployment on Edge Devices

This study presented a comparative analysis of different processor platforms for perception and path-planning tasks (Table 4). Experimental results indicated that the PC_GPU and Jetson_GPU achieved comparable detection performance, with bud and bloom detection counts of 4203 and 1423, respectively, demonstrating consistency between the two platforms. Regarding perception time, the PC_GPU attained an average preprocessing time of 0.2083 s and an average detection time of 0.0375 s, both substantially lower than those recorded for the Jetson_GPU, which were 0.5652 s and 0.0633 s, respectively. These findings suggested that the PC_GPU offered superior processing efficiency during the perception stage, primarily attributable to its enhanced computational and graphics processing capabilities. In contrast, as an embedded platform, the Jetson_GPU incurred greater processing delays in perception tasks due to constraints in power consumption and computational capacity, although it still largely satisfied real-time requirements.

**Figure 10:**
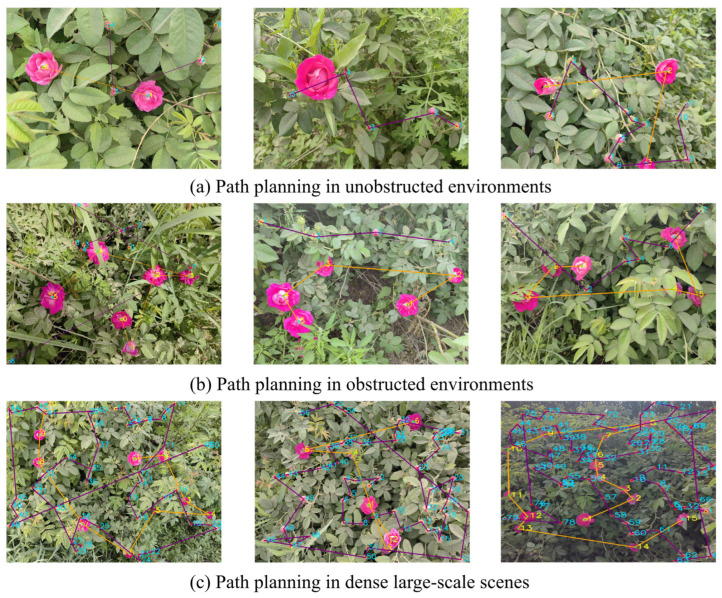
Path visualization results of the ROSE algorithm on edge devices.

Concerning path-planning performance, the PC_GPU achieved an average runtime of 0.004792 s, which was marginally lower than the 0.007108 s observed on the Jetson_GPU. Both platforms operated at the millisecond level and met real-time constraints. Given that both platforms processed the identical number of detection points, the planning algorithm generated logically consistent paths without significant deviation attributable to hardware variations. In summary, although the Jetson_GPU exhibited lower computational efficiency than the PC_GPU during the perception stage, it maintained high real-time performance and solution quality in the path-planning stage, thereby validating its effectiveness for deployment on edge devices. The visualized test results presented in Figure 10 further confirmed that, even under resource-constrained edge conditions, the proposed ROSE algorithm consistently generated continuous and feasible path trajectories that met practical operational requirements.

## 4. Discussion

The main contributions of this study lie in the proposal of a lightweight object detection model, termed Rose_YOLO, which improves the detection accuracy of rose buds and flowers in complex environments while substantially reducing the number of parameters and computational complexity through the introduction of the C2f-Faster-CGLU module and a novel Rose_Head detection head. In addition, an adaptive picking path-planning algorithm was developed by integrating a genetic algorithm with a reciprocating search strategy, enabling the dynamic selection of the optimal path-generation method according to scene complexity and thereby achieving an effective balance between picking efficiency and computational speed. In terms of the trade-off between model lightweightness and detection performance, the proposed Rose_YOLO demonstrates significant advantages over MWG-YOLO reported in the reference literature [31]. Specifically, Rose_YOLO contains only 1.58 M parameters, with a model size of 3.19 MB and a computational complexity of 4.6 GFLOPs, while achieving mAP@0.5 values of 96.6% and 91.7% for the Bloom and Bud categories, respectively. By comparison, although MWG-YOLO achieves a slightly higher overall mAP0.5 of 92.3%, it requires 8.86 M parameters, a model size of 17.3 MB, and 13.3 GFLOPs, all of which are substantially higher than those of the proposed model. While maintaining high detection accuracy, Rose_YOLO achieves a model compression rate of more than 46%, improves the detection accuracy for the Bud category by 3.8% over MWG-YOLO, and attains comparable accuracy for the Bloom category. These results demonstrate that Rose_YOLO achieves a superior synergy among lightweight deployment, computational efficiency, and detection performance, making it particularly suitable for resource-constrained agricultural edge-computing scenarios and therefore more promising for practical engineering applications.

Although the proposed method achieves satisfactory performance in bud and flower detection, several limitations remain. First, the detection module relies on two-dimensional image data, which can only provide the geometric center coordinates of targets in the image plane and lacks depth-related spatial information. As a result, it is difficult to accurately describe the distribution of rose buds and flowers in a real three-dimensional environment, which in turn affects the precision and robustness of picking path planning in complex field conditions. Second, the current dataset is mainly composed of rose samples collected under daytime and normal illumination conditions, and its coverage of diverse natural scenarios, such as rainy weather, dawn and dusk, and strong backlighting, remains insufficient. This limitation restricts the generalization capability of the model under complex climatic and illumination variations. In future work, we will integrate the proposed Rose_YOLO model and the ROSE algorithm into a real harvesting robotic platform. Specifically, this includes (1) acquiring three-dimensional spatial coordinates using a depth camera and incorporating a grasp pose estimation algorithm to map the recognition results of rose blooms and buds onto actual grasping actions; (2) designing a flexible gripping mechanism based on the different mechanical properties of rose blooms and buds to reduce harvesting damage; and (3) conducting field experiments to evaluate the stability of the harvesting process under varying lighting conditions. Meanwhile, it is observed that buds, being small targets, are densely distributed and frequently occluded by foliage, which hinders the model from stably extracting features. Although various lightweight improvements have been attempted, these designs primarily aim to enhance computational efficiency and overall mAP, without specifically strengthening feature extraction for small-target buds. As a result, the recall rate for buds has consistently remained around 82%. Future work will focus on incorporating attention mechanisms and data augmentation strategies to improve the recall rate of buds.

## 5. Conclusions

In conclusion, this study presents a novel coupled framework specifically designed for the selective harvesting of Hotan roses, successfully addressing the dual challenges of lightweight visual recognition and adaptive path planning. By introducing the RoseYOLO model, which integrates the C2f-Faster-CGLU module and a dual-path RoseHead detection head, we achieved a significant balance between accuracy and efficiency. The model demonstrated superior performance with a 96.6% mAP@0.5 for fully bloomed flowers and 91.7% for buds, while maintaining an extremely lightweight footprint of merely 3.19 MB and 1.58 M parameters. Furthermore, the proposed ROSE path-planning algorithm, combining an improved genetic algorithm with a reciprocating search mechanism, effectively optimized harvesting trajectories. It reduced the average path length to 2796.94 pixels and maintained a real-time computational efficiency of 0.00733 s, outperforming traditional methods like the ant colony algorithm and the basic genetic algorithm. However, limitations exist regarding the reliance on 2D imagery, which lacks depth information for precise 3D spatial reconstruction. Future work will focus on integrating depth sensors to acquire 3D coordinates and expanding the dataset to include diverse weather conditions (e.g., rain, dawn/dusk) to enhance the model’s robustness in unstructured agricultural environments.

## Figures and Tables

**Table 1:** Ablation experiments.

Baseline Model	C2f-Faster	C2f-Faster-CGLU	Rose_Head	Category	P (%)	R (%)	mAP@0.5	Model Size (MB)	Parameter	GFLOPs
YOLOv 8n	×	×	×	Bloom	91.0	92.3	96.4	5.94	3,006,038	8.1
				Bud	88.7	82.5	92.2			
YOLOv 8n	√	×	×	Bloom	90.0	91.3	96.1	4.62	2,300,838	6.3
				Bud	87.7	82.1	90.7			
YOLOv 8n	×	×	√	Bloom	88.6	91.9	96.2	4.81	2,362,453	6.5
				Bud	88.4	82.2	91.5			
YOLOv 8n	√	√	×	Bloom	91.2	93.2	95.5	4.44	2,222,930	6.2
				Bud	89.8	81.8	91.1			
YOLOv 8n	√	×	√	Bloom	90.3	92.1	96.7	3.55	175,723	4.9
				Bud	88.8	81.3	91.4			
YOLOv **8n**	**√**	**√**	**√**	**Bloom**	**90.4**	**91.6**	**96.6**	**3.19**	**1,579,345**	**4.6**
				**Bud**	**88.4**	**82.3**	**91.7**			

**Table 2:** Comparison of different models.

Model	Category	P (%)	R (%)	mAP@0.5	Model Size (MB)	Parameter	GFLOPs
YOLOv6n	Bloom	91.7	90.0	96.2	8.27	4,233,942	11.8
	Bud	85.2	84.0	90.6			
YOLOv8n	Bloom	91.0	92.3	96.4	5.94	3,006,038	8.1
	Bud	88.7	82.5	92.2			
YOLOv10n	Bloom	91.7	87.0	94.6	5.47	2,265,558	6.5
	Bud	81.0	86.0	90.9			
YOLOv11n	Bloom	91.4	91.7	96.9	5.25	2,582,542	6.3
	Bud	86.8	82.9	91.5			
**Rose_YOLO**	**Bloom**	**90.4**	**91.6**	**96.6**	**3.19**	**1,579,345**	**4.6**
	**Bud**	**88.4**	**82.3**	**91.7**			

**Table 3:** Comparison of different path-planning algorithms.

Planning Strategy	Number of Detection Points		Average Runtime (s)	Average Distance (Pixel)
	Bud	Bloom		
Reciprocating Algorithm	6114	1816	0.000205	10,411.54
Ant Colony Optimization	6114	1816	1.980572	3827.24
Genetic Algorithm	6114	1816	0.219869	5778.46
**ROSE Algorithm**	**6114**	**1816**	**0.00733**	**2796.94**

**Table 4:** Deployment results on edge devices.

Processor	Number of Detection Points		Perception Time(s)		Path-Planning Performance(s)
	Bud	Bloom	Average Preprocessing Time	Average Detection Time	Average Runtime
PC_GPU	4203	1423	0.2083	0.0375	0.004792
Jetson_GPU	4203	1423	0.5652	0.0633	0.007108

## Data Availability

The original contributions presented in this study are included in the article; further inquiries can be directed to the corresponding author.
